# HLA‐DR polymorphism in SARS‐CoV‐2 infection and susceptibility to symptomatic COVID‐19

**DOI:** 10.1111/imm.13450

**Published:** 2022-03-08

**Authors:** Stuart Astbury, Catherine J. Reynolds, David K. Butler, Diana C. Muñoz‐Sandoval, Kai‐Min Lin, Franziska P. Pieper, Ashley Otter, Afroditi Kouraki, Lola Cusin, Jessica Nightingale, Amrita Vijay, Simon Craxford, Guruprasad P. Aithal, Patrick J. Tighe, Joseph M. Gibbons, Corinna Pade, George Joy, Mala Maini, Benny Chain, Amanda Semper, Timothy Brooks, Benjamin J. Ollivere, Áine McKnight, Mahdad Noursadeghi, Thomas A. Treibel, Charlotte Manisty, James C. Moon, COVIDsortium Investigators*, Hakam Abbass, Aderonke Abiodun, Mashael Alfarih, Zoe Alldis, Daniel M. Altmann, Oliver E. Amin, Mervyn Andiapen, Jessica Artico, João B. Augusto, Georgiana L. Baca, Sasha N. L. Bailey, Anish N. Bhuva, Alex Boulter, Ruth Bowles, Rosemary J. Boyton, Olivia V. Bracken, Ben O’Brien, Tim Brooks, Natalie Bullock, David K. Butler, Gabriella Captur, Nicola Champion, Carmen Chan, Aneesh Chandran, David Collier, Jorge Couto de Sousa, Xose Couto‐Parada, Teresa Cutino‐Moguel, Rhodri H. Davies, Brooke Douglas, Cecilia Genova, Keenan Dieobi‐Anene, Mariana O. Diniz, Anaya Ellis, Karen Feehan, Malcolm Finlay, Marianna Fontana, Nasim Forooghi, Celia Gaier, Joseph M. Gibbons, Derek Gilroy, Matt Hamblin, Gabrielle Harker, Jacqueline Hewson, Lauren M. Hickling, Aroon D. Hingorani, Lee Howes, Alun Hughes, Gemma Hughes, Rebecca Hughes, Ivie Itua, Victor Jardim, Wing‐Yiu Jason Lee, Melaniepetra Jensen, Jessica Jones, Meleri Jones, George Joy, Vikas Kapil, Hibba Kurdi, Jonathan Lambourne, Kai‐Min Lin, Sarah Louth, Mala K. Maini, Vineela Mandadapu, Charlotte Manisty, Áine McKnight, Katia Menacho, Celina Mfuko, Oliver Mitchelmore, Christopher Moon, James C. Moon, Diana C. Muñoz‐Sandoval, Sam M. Murray, Mahdad Noursadeghi, Ashley Otter, Corinna Pade, Susana Palma, Ruth Parker, Kush Patel, Babita Pawarova, Steffen E. Petersen, Brian Piniera, Franziska P. Pieper, Daniel Pope, Mary Prossora, Lisa Rannigan, Alicja Rapala, Catherine J. Reynolds, Amy Richards, Matthew Robathan, Joshua Rosenheim, Genine Sambile, Nathalie M. Schmidt, Amanda Semper, Andreas Seraphim, Mihaela Simion, Angelique Smit, Michelle Sugimoto, Leo Swadling, Stephen Taylor, Nigel Temperton, Stephen Thomas, George D. Thornton, Thomas A. Treibel, Art Tucker, Jessry Veerapen, Mohit Vijayakumar, Sophie Welch, Theresa Wodehouse, Lucinda Wynne, Dan Zahedi, Daniel M Altmann, Rosemary J. Boyton, Tim Brooks, Benjamin Chain, Mala K. Maini, Charlotte Manisty, Áine McKnight, James C. Moon, Mahdad Noursadeghi, Thomas A. Treibel, Guruprasad P. Aithal, Waheed Ashraf, Stuart Astbury, Jonathan K. Ball, Joseph G. Chappell, Simon Craxford, Lola M. L. Cusin, Joshua D. Duncan, Adeel Ikram, William L. Irving, Hannah J. Jackson, Anthony Kelly, Melanie Lingaya, Ben A. Marson, Jayne Newham, Jessica Nightingale, Alan Norrish, Barbara Nowicka, Benjamin J. Ollivere, Alexander W. Tarr, Patrick J. Tighe, Theocharis Tsoleridis, Richard A. Urbanowicz, Ana M. Valdes, Amrita Vijay, Ana M. Valdes, Rosemary J. Boyton, Daniel M. Altmann

**Affiliations:** ^1^ NIHR Nottingham Biomedical Research Centre Nottingham University Hospitals NHS Trust and the University of Nottingham Nottingham UK; ^2^ Nottingham Digestive Diseases Centre School of Medicine University of Nottingham Nottingham UK; ^3^ Department of Infectious Disease Imperial College London London UK; ^4^ National Infection Service Public Health England Porton Down UK; ^5^ Division of Rheumatology, Orthopaedics and Dermatology School of Medicine University of Nottingham Nottingham UK; ^6^ School of Life Sciences University of Nottingham Nottingham UK; ^7^ Barts and the London School of Medicine and Dentistry Blizard Institute Queen Mary University of London London UK; ^8^ Barts Heart Centre St. Bartholomew's Hospital London UK; ^9^ Division of Infection and Immunity University College London London UK; ^10^ Institute of Cardiovascular Sciences University College London London UK; ^11^ Lung Division Royal Brompton and Harefield Hospitals Guy’s and St Thomas’ NHS Foundation Trust London UK; ^12^ Department of Immunology and Inflammation Imperial College London London UK

**Keywords:** COVID‐19, HLA, immunogenetics, SARS‐CoV‐2, T‐cell immunity, vaccine

## Abstract

SARS‐CoV‐2 infection results in different outcomes ranging from asymptomatic infection to mild or severe disease and death. Reasons for this diversity of outcome include differences in challenge dose, age, gender, comorbidity and host genomic variation. Human leukocyte antigen (HLA) polymorphisms may influence immune response and disease outcome. We investigated the association of HLAII alleles with case definition symptomatic COVID‐19, virus‐specific antibody and T‐cell immunity. A total of 1364 UK healthcare workers (HCWs) were recruited during the first UK SARS‐CoV‐2 wave and analysed longitudinally, encompassing regular PCR screening for infection, symptom reporting, imputation of HLAII genotype and analysis for antibody and T‐cell responses to nucleoprotein (N) and spike (S). Of 272 (20%) HCW who seroconverted, the presence of HLA‐DRB1*13:02 was associated with a 6·7‐fold increased risk of case definition symptomatic COVID‐19. In terms of immune responsiveness, HLA‐DRB1*15:02 was associated with lower nucleocapsid T‐cell responses. There was no association between DRB1 alleles and anti‐spike antibody titres after two COVID vaccine doses. However, HLA DRB1*15:01 was associated with increased spike T‐cell responses following both first and second dose vaccination. Trial registration: NCT04318314 and ISRCTN15677965.

## Graphical abstract

SARS‐CoV‐2 infection results in different outcomes ranging from asymptomatic infection, to mild or severe disease and death. We investigated the association of HLAII alleles with case definition symptomatic COVID‐19, virus‐specific antibody and T cell immunity. Presence of HLA‐DRB1*13:02 was associated with increased risk of symptomatic COVID‐19, while, after vaccination, HLA DRB1*15:01 was associated with increased spike T cell responses.

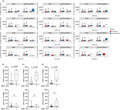

## INTRODUCTION

Infection by SARS‐CoV‐2 leads to diverse outcomes in different individuals, the determinants of such variability encompassing factors such as age, gender, obesity and host genetics. A number of loci have already been implicated in genetic susceptibility, many proposed to impact on innate immune mechanisms [1, 2, 3, 4]. In terms of adaptive immunity, for many infectious diseases, there is a strong impact of human leukocyte antigen (HLA) polymorphisms, since this complex contains the key immune response genes determining peptide presentation to T cells [5, 6, 7]. Effects may be apparent in outcomes, from susceptibility to infection, disease severity, disease progression, antibody titre or magnitude of T‐cell response. Effects of this type are seen in HLA‐associated differential outcomes following infection by HIV, HBV, HCV, HPV and *Mycobacterium tuberculosis*, among many others [5, 6, 7, 8, 9, 10]. While relatively few cases have been mapped to the level of specific HLA‐peptide interactions, the presumed mechanism for such HLA association is that the peptide‐binding grooves of particular alleles may better present key, immunogenic epitopes to protective T cells [11]. Such effects can be even more apparent in differential responsiveness to vaccines: profound differences associated with HLA type are seen in antibody titre following vaccination for influenza, measles, anthrax and HBV [12, 13. In the case of HBV, for example, HLA‐DRB1 polymorphisms are involved in vaccine non‐responsiveness. The HLA complex encompasses more than 250 expressed genes, and infectious disease associations have been noted to different loci, in line with implicated immune mechanisms [14]. For example, different aspects of HIV susceptibility highlight the role of HLAII interactions with CD4 T cells, of HLAI interactions with CD8, and of HLA‐B and C products with KIR on NK cells [7]

We here consider the question of HLAII association with outcome following natural infection by SARS‐CoV‐2 and COVID‐19 vaccination in a well‐documented cohort of frontline healthcare workers (HCWs) at UK hospitals in London and Nottingham [15, 16, 17, 18, 19, 20, 21], studied longitudinally by repeat PCR‐testing and serology since UK lockdown in March 2020. HCW are at higher SARS‐CoV‐2 infection risk [22, 23, 24] with reported estimates from 3·4 to 18 times higher than the general population [23, 24, 25]. As in the general population, the majority of SARS‐CoV‐2 infections tracked in our HCW cohorts are mild or asymptomatic, allowing investigation of the range of immune responses in COVID‐19 from case definition symptoms to atypical symptoms and asymptomatic infection [15, 16, 17, 18, 19, 20, 21]. Data previously reported from this HCW cohort indicate that antibody and T‐cell responses in natural infection can be variable and discordant and with antibody responses starting to wane over the first 6‐months from initial infection [17, 18, 19]. T‐cell responses tended to be higher in male infected HCW and those reporting case‐definition symptoms. Neutralizing antibody responses tended to be higher in older women [19]. We here investigate the hypothesis that HLAII polymorphisms influence outcome in SARS‐CoV‐2 infection in terms of likelihood of infection, symptomatic disease, antibody response and T‐cell response. While noting that it would be of value also to consider potential contributions of protective CD8 responses and HLAI polymorphisms, the present study was based on the premise of a central axis of adaptive immunity operating through CD4 T cells and generation of antibody, using analysis of CD4 and antibody responses as we have previously described [19, 20, 21]. Another recent study has focused on the potential role of HLAI‐associated, protective CD8 responses: nucleoprotein 105‐113/B*07:02‐specific T‐cell responses were associated with mild disease and antiviral protection through a sustained repertoire of high avidity CD8 T cells [26]. We have here considered CD4 and antibody immune responses following natural infection and after first and second doses of the Pfizer BNT162b2 vaccine in SARS‐CoV‐2 naïve and previously infected vaccinees.

## MATERIALS AND METHODS

### HCW cohorts

A 5‐hospital HCW longitudinal study (n = 1364) of UK first wave SARS‐CoV‐2 infection consisting of two initially independent studies (PANTHER, Nottingham: Nottingham City Hospital and Queen's Medical Centre, part of Nottingham University Hospitals NHS trust; COVIDsortium, London: St Bartholomew's, Nightingale and Royal Free Hospitals) that methodologically aligned in April 2020 (NCT04318314). London ethical approval was South Central, Oxford A Research Ethics Committee, reference 20/SC/0149. Nottingham was initially under a Human Tissue Authority licence in Nottingham (Licence number: 11035) and subsequently North‐West – Greater Manchester South Research Ethics Committee, reference 20/NW/0395. A detailed description of both cohorts can be found elsewhere [15, 16, 17, 18, 19, 20, 21].

The subset of participants included for the post vaccination part of the study and recruitment criteria are detailed in Figure S1.

### SARS‐CoV‐2 serology

Both studies performed serial SARS‐CoV‐2 serology testing assessing antibodies to both spike (S1) and nucleoprotein (N). The London samples were analysed using commercial assays; the Euroimmun anti‐SARS‐CoV‐2 enzyme‐linked immunosorbent assay (ELISA) targeting IgG specific for S1 [27] and the Roche Elecsys Anti‐SARS‐CoV‐2 electrochemiluminescence immunoassay (ECLIA) that detects antibodies (including IgG) for N protein. Anti‐RBD antibodies were detected using the quantitative Roche Elecsys^®^ anti‐SARS‐CoV‐2 ECLIA spike assay (Roche ACOV2S, Product code: 09289275190). These were undertaken at the Rare and Imported Pathogens Laboratory at Public Health England using standard protocols. Positive was defined as (Euroimmun) a ratio >1·1, and (Roche) a electrochemiluminescence sample to lot‐specific cut‐off index >1, as per manufacturers’ instructions. Reported assay sensitivity (92·3% and 96·2%–100% for Roche and Euroimmune, respectively) and specificities (100%) are high [28].

For all sera collected in 2020 and the first dose of the vaccine, the Nottingham study used in‐house robotically delivered ELISAs cross‐validated by the same Public Health England laboratory (PHE, Porton‐Down, UK). In brief, they were ELISAs to S1 and N protein detecting IgG. Individuals were classified as seropositive if they had a positive titre to either at any time point. Seropositivity was defined as samples where the average measurement of the duplicates exceeded 2× the median value for the pooled negative controls. Samples higher than the highest negative, but lower than or equal to 2× the median of the pooled negatives were deemed indeterminate for COVID‐19. For the second dose of the vaccine, the same methods and laboratory were used as those for the London cohort described above.

### Symptom definition

Healthcare workers were classified as having case‐definition symptoms if at any time point they self‐reported the following symptoms (fever, dry cough, loss of sense of smell or taste) using the symptoms‐based model developed previously [29], or if they had to self‐isolate due to symptoms of COVID‐19.

### Sample genotyping

Samples were genotyped using the Illumina Infinium Global Screening Array‐24v1+MD, quality control and filtering (relatedness, heterozygosity, sample and SNP call rate) was carried out in PLINK v1.90b6.12 [30] HLA alleles A, B, C, DQA1, DQB1, DPB1, DRB1 were imputed using the HLA Genotype Imputation with Attribute Bagging (HIBAG) v1.24.0 package running in R v4.0.1 [31]. HLA and SNP genotypes from the publicly available HLARES and HapMap Phase 2 datasets, genotyped using the same array as the input data, were used as references for imputation. Initially a multi‐ethnic panel was used, and where appropriate, ethnicity specific reference panels based on individuals of African, Asian and European descent were used to increase imputation accuracy.

### T‐cell response analysis

Peripheral blood mononuclear cells (PBMC) and serum was isolated and stored as previously described [19, 20, 21]. T‐cell ELISpot analysis was carried out using pre‐coated ELISpot plates (Mabtech 3420‐2APT), read on an AID classic ELISpot plate reader (Autoimmun Diagnostika GMBH) and analysed as previously reported [19, 20, 21].

### Statistical analysis

Associations between DRB1 alleles and binary outcomes (Covid‐19 case definition symptoms, seropositivity) were assessed by standard logistic regression. Association with quantitative outcomes were assessed by unpaired *t*‐tests if assumptions of normality held, otherwise by Mann–Whitney tests. Data for antibody titres were normalized to a mean of 0 and variance of 1 for each cohort and data were meta‐analysed using a Mantel Hanzel model. All analyses were carried out using Prism GraphPad 8.0 and StatsDirect 3.0.

#### Adjustment for multiple comparisons

We considered statistically significant *P*‐values *p* < 0·0025 adjusting for 15 DRB1 allele tests (alleles with carrier frequencies >1·5%, which comprise DRB1*04:05, DRB1*16:01, DRB1*15:02, DRB1*01:02, DRB1*04:07, DRB1*12:01, DRB1*08:01, DRB1*11:04, DRB1*04:02, DRB1*13:02, DRB1*04:04, DRB1*04:04, DRB1*14:01, DRB1*13:01, DRB1*01:01, DRB1*11:01, DRB1*04:01, DRB1*15:01, DRB1*03:01 and DRB1*07:01).

#### Statistical power

The analyses carried out had 80% power to detect associations between DRB1 alleles with *P* < 0·0025 (adjusting for 15 DRB1 allele comparisons) between seropositivity (total *n* = 1365) and DRB1 alleles freq 1% of higher with odds ratios of 3·75, for DRB1 alleles with allele freq 5% with odds ratios of 2·1 or higher and for DRB1 freq 10% odds ratios (ORs) of 1·75 for associations with symptoms among seropositive individuals allele freq 2·5% with OR ≥ 4·75, for freq 5% OR 3·4, for freq 10% OR 2·6 (total *n* = 265) for post vaccination titres (*n* = 432) for differences of 1 or more standard deviations between mean log titre levels alleles with allele frequencies of 1% or higher, and for differences of 0·44 SDs between alleles where the SD for log10 titre levels is 0·42 and average post vaccination log10 titre levels are 4·1.

## RESULTS

We initially explored the extent to which HLA‐DRB1 alleles are associated with symptomatic COVID‐19 in seropositive HCW following SARS‐CoV‐2 infection. The London COVIDsortium (*n* = 731) and Nottingham PANTHER (*n* = 633) cohorts were recruited during the first wave in the UK and 20% of HCW seroconverted (Figure S1, Table S1). There was no difference in HLA‐DRB1 frequency between seropositive (*n* = 272) and seronegative (*n* = 1092) individuals where seropositivity refers to IgG positive titres for either nucleoprotein or spike S1 (Figure S2a). The London and Nottingham cohorts were analysed separately since the serology had been measured using different assays.

**FIGURE 1:**
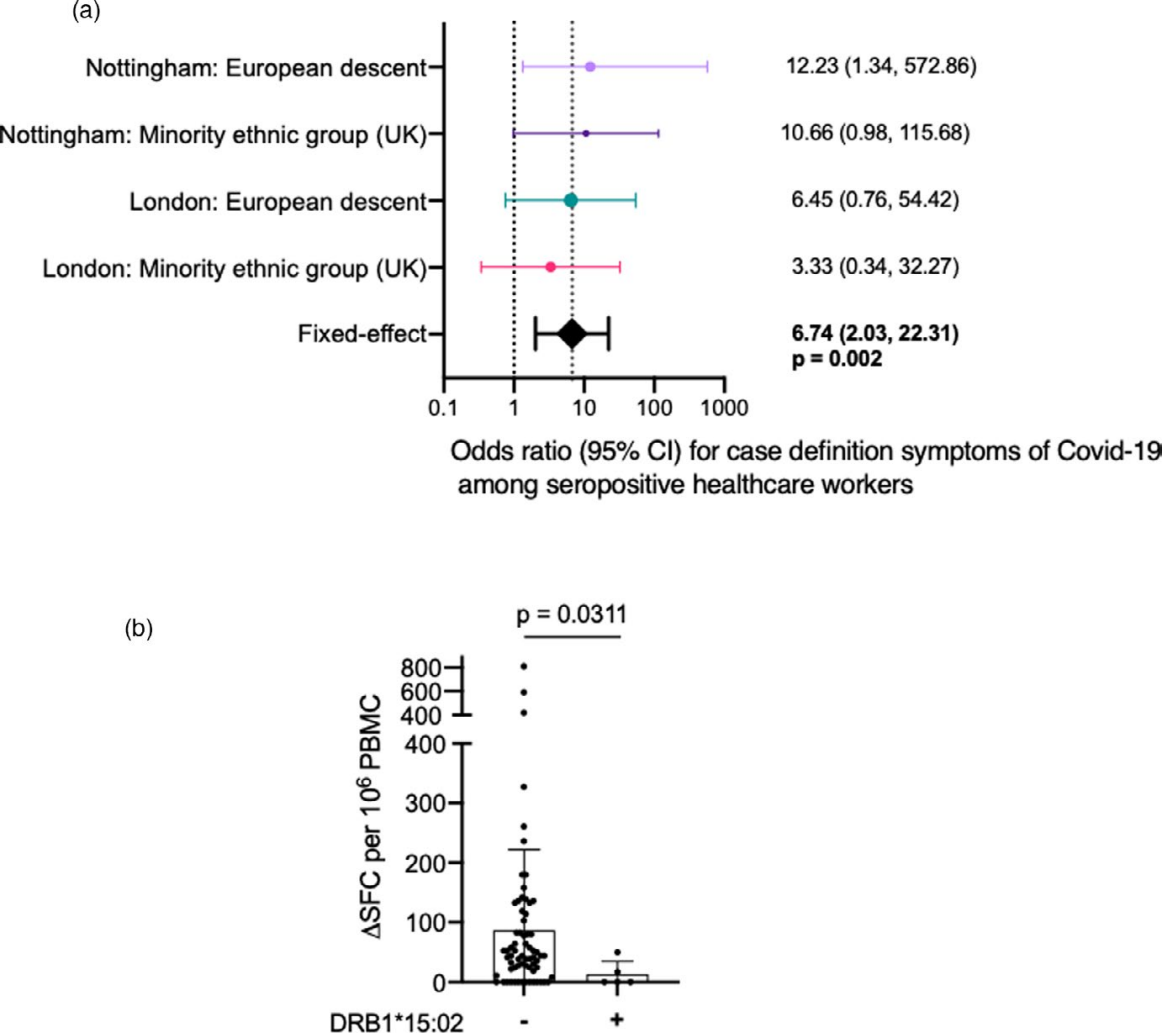
Association between HLA DRB1 alleles, the presence of case definition symptoms and T cell immune responses to SARS‐CoV‐2 following natural infection: (a) consistent association of DRB1*13:02 with the presence of case definition symptoms; (b) Association between the absence of HLA‐DRB1*15:02 and T‐cell responses against nucleoprotein peptide pool (HLA‐DRB1*15:02 −, *n* = 68, HLA‐DRB1*15:02 +, *n* = 5). Bars show mean with SD. *P* value calculated using a Mann–Whitney *U* test. PBMC for T cell assays were taken on average 121 (range 71–174) days following first presentation of case definition symptoms. HLA, human leukocyte antigen; PBMC, peripheral blood mononuclear cells

Although the HCW cohort study did not detect population‐level effects of HLA polymorphism on SARS‐CoV‐2 infection per se, within seropositive individuals there was an association between carrying HLA‐DRB1*13:02 and the presence of self‐reported case‐definition symptoms (Figure S2b). Expression of HLA‐DRB1*13:02 is associated with an increased chance of suffering symptomatic disease in infected individuals. Results from both cohorts (London and Nottingham) split broadly by ethnicity (self‐reported European descent vs. Minority ethnic group [UK]) showed DRB1*13:02 to be significantly associated with higher odds of a seropositive individual presenting case‐definition symptoms (OR = 6·74, 95% confidence interval 2·03–22·31; *P* = 0·002) (Figure 1a). The data suggest greater susceptibility to symptomatic disease in HLA‐DRB1*13:02 individuals.

We next considered whether HLAII impacted on the magnitude of the T‐cell response to S or N in infected HCW. We have previously reported T‐cell ELISpot responses against SARS‐CoV‐2 in the London HCW cohort [19, 20, 21]. Since T‐cell analysis was conducted in a smaller sample, we interpret these findings with caution. We did not observe strong associations with T‐cell responses that could pass a multiple test correction (*P* < 0·0025) but found a nominal association between lower responses to the N among carriers of DRB1*15:02 (Figure 1b). In general, infected HLA‐DRB1*15:02 HCW in this cohort tended to cluster at the lower end of T‐cell responsiveness to both spike and nucleoprotein, often making little or no T‐cell response after infection (Figure S3).

There was no significant association between HLA‐DRB1 alleles and antibody titre after the first vaccine dose in HCW with no prior SARS‐CoV‐2 infection (Figure S4a). However, we and others have previously shown that there is a strong and significant immune boosting effect of prior COVID‐19 infection conferred on first vaccine dose [20, 21, 32, 33. In the present study, significant negative associations between S antibody titres after one dose of the BNT162b2 vaccine and HLA DRB1 alleles DRB1*04:04 and DRB1*07:01 were observed among single dose vaccinated individuals with prior SARS‐CoV‐2 infection, whilst DRB1*03:01 was associated with significantly higher anti‐S titres (Figure S4b); this was not apparent in SARS‐CoV‐2 naïve vaccinees. After two doses of vaccine, none of these DRB1 allele associations remain significant, arguing that HLAII polymorphisms do not substantially impact antibody responses to COVID‐19 vaccination.

**FIGURE 2:**
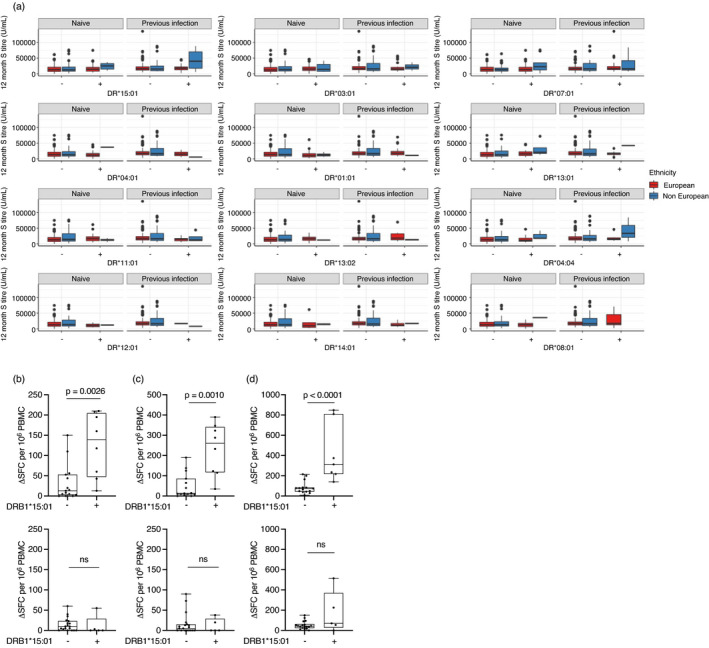
HLA DRB1 alleles not associated with enhanced antibody responses but DR15:01 associated with higher T cell responses to spike in prior SARS‐CoV‐2 infected HCW: (a) Anti‐spike titres after two doses of COVID vaccine were evaluated in the context of the top 12 most frequent DR alleles in HCW from the COVIDsortium (*n* = 251) and PANTHER (*n* = 169) cohorts. (b–d) Association between the presence of the DRB1*1501 allele and T‐cell responses against (b) spike protein in single dose vaccinated HCW, (c) spike peptide pool in single dose vaccinated HCW and (d) spike peptide pool in two dose vaccinated HCW with prior SARS‐Co‐V‐2 infection (upper panel, *n* = 23) and SARS‐CoV‐2 naïve vaccinees (lower panel, *n* = 23). *P* values were calculated using a Mann‐Whitney *U* test. Data are shown as box and whisker plots. HCW, healthcare worker; HLA, human leukocyte antigen

We then explored whether a similar pattern of HLAII associated enhancement was seen in T‐cell responses to S in SARS‐CoV‐2 naïve and prior infected HCW after one or two doses of vaccine (Figure 2b–d). DRB1*15:01 carriers showed a 4–6‐fold enhancement of T‐cell responses against S compared to non‐DRB1*15:01 carriers (Figure 2b). This observation was only apparent in the context of vaccinees with prior SARS‐CoV‐2 infection, and no difference was observed among SARS‐CoV‐2 naïve HCW (Figure 2b–d).

## DISCUSSION

In this study our high‐granularity, longitudinal analysis of large HCW cohorts has allowed an initial appraisal of HLAII allelic effects in diverse aspects of susceptibility to infection and symptoms as well as specific immune responses. Our comments carry the caveat that this sample size is relatively small from which to draw firm conclusions when one considers the number of distinct HLAII heterozygous combinations present. Nevertheless, there may be some interesting leads for further analysis. A further limitation of the study is that, although the demographic characteristics of our study sample (socioeconomic status, ethnicity, body mass index, age) cover a broad spectrum of the UK population, the exposure to SARS‐CoV‐2 in this group is likely to be higher, and hence findings may not be readily extrapolated.

For a new human pathogen that has spread across the globe so effectively and rapidly, one might perhaps not expect to find explicit examples of differences in resistance to infection, and this was indeed the case. While our cohort study did not detect population‐level effects of HLAII polymorphism on SARS‐CoV‐2 infection, within seropositive individuals there was an association between presence of HLA‐DRB1*13:02 and symptomatic disease. Among the many research challenges posed by the COVID‐19 pandemic has been decoding the differential pathophysiology of diverse outcomes following exposure, from asymptomatic presentation to mild disease, severe disease or death. Our findings place differential immune response gene effects of HLAII sequence peptide presentation within that mechanistic landscape. Notwithstanding our immunological analysis of the HCW cohort, it remains to be seen whether increased symptomatic disease in HLA‐DRB1*13:02 individuals relates either to inadequacy of a protective antiviral response, or to a differential immunopathogenic contribution to symptomology in these individuals. Interestingly, this allele is implicated in other examples of differential outcome after viral infection, notably, protection against persistent HBV infection [34]. The allele is found in populations across the globe, though common (approaching one in five) in some populations including Saudi Arabia, South Korea and Rwanda (www.allelefrequencies.net).

While specific differences in SARS‐CoV‐2 epitope specific responses did not reach significance with respect to HLADRB1*13:02, we observed an effect on T‐cell responsiveness of the allelic variants of HLA‐DR15, that is, HLA‐DRB1*15:01 and HLA‐DRB1*15:02. HLA‐DR15 sequences encompass multiple alleles preferentially represented in populations inhabiting different regions of the world: HLA‐DRB1*15:01 is more frequent in individuals of European Caucasian origin, while HLA‐DRB1*15:02 is the predominant HLA‐DR15 allele in Eastern and Southeastern Asia (www.allelefrequencies.net). The alleles differ by a single amino acid at position 86β, this impacting both peptide binding specificity, heterodimeric stability and presentation to CD4 cells [35, 36, 37, 38, 39]. From our analysis, HLA‐DRB1*15:01 individuals tend to cluster at the higher end of T‐cell responses, HLA‐DRB1*15:02 individuals at the lower end. This enhanced responsiveness in HLA‐DRB1*15:01 individuals extended to the boosted responses that we have previously described in people vaccinated following a prior natural infection [20, 21. Thus, assuming a classic ‘high‐responder’ immune response gene effect through ability of the HLA‐DRB1*15:01 binding groove to present specific spike epitopes, it is assumed that the epitope(s) in question must be immunodominant and processed for presentation both during infection and vaccination, and thus visualized as part of hybrid‐immunity boosting. The number of DRB1*15:02+ individuals in our study is fairly modest, and our results regarding this allele should be seen as hypothesis‐generating, requiring further confirmation.

In conclusion, HLAII polymorphisms exert an effect on presence of symptoms in natural SARS‐CoV‐2 infection. However, we found no evidence for a role in seroprevalence following infection. The magnitude of spike antibody response is also unaffected by DRB1 genotype. However, some HLA‐DRB1 alleles are associated with enhanced or muted post natural infection and vaccination T‐cell responses. Our findings suggest that, as management of the COVID‐19 pandemic moves into a phase where there is demand for a more nuanced understanding of differences in protective immunity, especially the issue of understanding vulnerable groups and the targeting of booster vaccines, there will be a role for determination of immunogenetic risk factors.

## CONFLICT OF INTEREST

R.J.B. and D.M.A. are members of the Global T cell Expert Consortium and have consulted for Oxford Immunotec outside the submitted work.

## AUTHOR CONTRIBUTIONS

Rosemary J. Boyton, Daniel M. Altmann and Ana M. Valdes conceptualized the study reported. Rosemary J. Boyton and Daniel M. Altmann designed and supervised the T‐cell experiments. Catherine J. Reynolds, David K. Butler, Diana C. Muñoz‐Sandoval, Kai‐Min Lin, and Franziska P. Pieper performed the T‐cell experiments. Stuart Astbury and Ana M. Valdes carried out HLA imputation and statistical analysis. Timothy Brooks and Amanda Semper supervised S1 IgG and N IgG/IgM studies. Ashley Otter and Amanda Semper analysed the RBD and N antibody assays. Patrick J. Tighe and Lola Cusin carried out antibody testing in Nottingham cohort. Catherine J. Reynolds, David K. Butler, Diana C. Muñoz‐Sandoval, Kai‐Min Lin, Franziska P. Pieper, Joseph M. Gibbons, Corinna Pade and COVIDsortium investigators processed samples. George Joy coordinated HCW recruitment and organized the cohort database for the London cohort. Timothy Brooks, Charlotte Manisty, Áine McKnight, Thomas A. Treibel, James C. Moon, and Mahdad Noursadeghi established the COVIDsortium HCW cohort. Ana M. Valdes, Benjamin J. Ollivere and Guruprasad P. Aithal established the Nottingham PANTHER cohort. Rosemary J. Boyton, Thomas A. Treibel, James C. Moon, and Charlotte Manisty designed the vaccine sub‐study. Catherine J. Reynolds, Stuart Astbury, Rosemary J. Boyton, Daniel M. Altmann and Ana M. Valdes analysed the data. Daniel M. Altmann, Catherine J. Reynolds, Stuart Astbury, Áine McKnight, Mala Maini, Benny Chain, Charlotte Manisty, Thomas A. Treibel, James C. Moon, Amanda Semper, Timothy Brooks, Mahdad Noursadeghi, Ana M. Valdes and Rosemary J. Boyton interpreted the data. Rosemary J. Boyton, Daniel M Altmann and Ana M. Valdes wrote the manuscript. All the authors reviewed and edited the manuscript and figures.

## Supporting information

Supplementary Material

## Data Availability

All data needed to evaluate the conclusions in the paper are present in the paper or the Supplementary Materials.
