# Supplementary material for: HLA‐DR polymorphism in SARS‐CoV‐2 infection and susceptibility to symptomatic COVID‐19
Source: Immunology. 2022 Mar 8;166(1):68–77. doi: 10.1111/imm.13450 (PMC9111350; doi:10.1111/imm.13450)
Supplement: Supplementary file 1 — Supplementary Material [file IMM-166-68-s001.docx]

**Supplementary Materials**

**Supplementary Methods**

**Supplementary Table**

Supplementary Table S1. Descriptive characteristics and SARS-CoV-2 seropositivity data for the COVIDsortium (London) and PANTHER (Nottingham) HCW cohorts.  ITU= Intensive Therapy Unit; PPE = Personal Protective Equipment

**Supplementary Figures**

Supplementary Figure S1. Consort Diagram for both cohorts (A) COVIDsortium cohort (London) and (B) PANTHER cohort (Nottingham)

Supplementary Figure S2. DRB1 alleles and (A) seropositivity in HCW; (B) presence of case definition COVID-19 symptoms among seropositive healthcare workers

Supplementary Figure S3. Cumulative magnitude of the T cell response to spike (red) and N (blue) protein ordered by increasing magnitude of response in HCW with laboratory-confirmed SARS-CoV-2 infection (n=70)

Supplementary Figure S4. Association between DRB1 alleles and serological responses (IgG to spike S1) in COVIDsortium (London) and PANTHER (Nottingham) cohorts combined meta-analyses

**Supplementary Methods**

**Nottingham cohort serology**. Serum samples were serially diluted in 3% skimmed milk powder in PBS containing 0.05% Tween 20 and 0.05% sodium azide. All assays were performed on Biotek Precision liquid handling robots in a class II microbiological safety cabinet. For endpoint dilution ELISAs, sera were progressively 4-fold diluted from 1:150 to 1;38,400. ELISA was performed by coating 384 well Maxisorp (NUNC) assay plates with either 20 µL per well of 0.5 µg.mL^-1^ of Wuhan strain SARS-CoV-2 spike protein S1 subunit (His tagged, HEK293 expressed; Sino Biological) or SARS-CoV-2 nucleocapsid (His Tagged, baculovirus expressed; Sino Biological) in carbonate-bicarbonate buffer (CBC; Merck), or human IgG at 1 µg.mL^-1^ in CBC buffer as controls. Plates were sealed with foil film and incubated overnight at 4 °C. Plates were then washed with PBS with 0.05% Tween 20 (PBS-T) 3 times using a ThermoFisher Wellwash Versa plate washing robot. Wells were immediately filled with 100 µL of 3% skimmed milk powder (w/v) in PBS and 0.05% sodium azide (PBS-MA) and blocked overnight at 4 °C. Assay plates were then washed 3 times and 20 µL of pre-diluted serum sample (including SARS-CoV-2 antibody-positive and negative serum controls) added in duplicate wells. After one hour at 21 °C, the plate was washed 3 times in PBS-T, followed by addition of 20 µL of gamma chain-specific anti-human IgG-HRP conjugate (Sigma A0170-1ML)  at  1:30,000 dilution, incubating for one hour at 21 °C. Following a final three washes with PBST, 40 µL One-step UltraTMB substrate solution (ThermoScientific) was added to each well. After incubating for 20 minutes at room temperature, 40 µL of 2N H_2_SO_4_ was added to each well and Absorbance was measured at 450nm using a GlowMax Explorer microplate reader (Promega).

Seropositivity where determined as samples where the average measurement of the duplicates exceeds 2x the Median for the pooled negative controls. Samples higher than the highest negative, but lower than or equal to 2x the median of the pooled negatives were deemed indeterminate for covid19.

**Supplementary Table**

Supplementary Table S1. Descriptive characteristics and SARS-CoV-2 seropositivity data for the COVIDsortium (London) and PANTHER (Nottingham) HCW cohorts.  ITU= Intensive Therapy Unit; PPE = Personal Protective Equipment

|  |  | **London** | |  | **Nottingham** | |  |
| --- | --- | --- | --- | --- | --- | --- | --- |
|  |  | **Ab-** | **Ab+** | **Seropositive%** | **Ab-** | **Ab+** | **Seropositive%** |
| **Age** | **Mean years** | 37.8 | 39.4 |  | 43.1 | 43.9 |  |
|  |  |  |  |  |  |  |  |
|  | **(SD)** | 10.9 | 11 |  | 11.6 | 11.6 |  |
|  |  |  |  |  |  |  |  |
| **Sex** | **M** | 188 | 54 | 22.3% | 115 | 22 | 16.1% |
|  | **F** | 384 | 102 | 21.0% | 401 | 93 | 18.7% |
|  |  |  |  |  |  |  |  |
| **Covid-19**  **symptoms** | **Yes** | 111 | 71 | 39.0% | 108 | 42 | 28.0% |
|  | **No** | 463 | 86 | 15.7% | 410 | 73 | 15.1% |
|  |  |  |  |  |  |  |  |
| **Ethnicity** | **Minority ethnic group (UK)** | 327 | 77 | 23.55% | 127 | 40 | 31.49% |
|  | **European descent** | 372 | 107 | 22.3% | 439 | 87 | 16.5% |
|  |  |  |  |  |  |  |  |
| **Use of PPE** | **ITU role** | 109 | 17 | 13.5% | 36 | 1 | 2.7% |
|  | **use PPE not ITU** | 353 | 112 | 24.1% | 337 | 84 | 20.0% |
|  | **other roles** | 112 | 28 | 20.0% | 147 | 30 | 16.9% |

**Supplementary Figures**

Supplementary Figure S1. Consort Diagram for both cohorts (A) COVIDsortium cohort (London) and (B) PANTHER cohort (Nottingham)


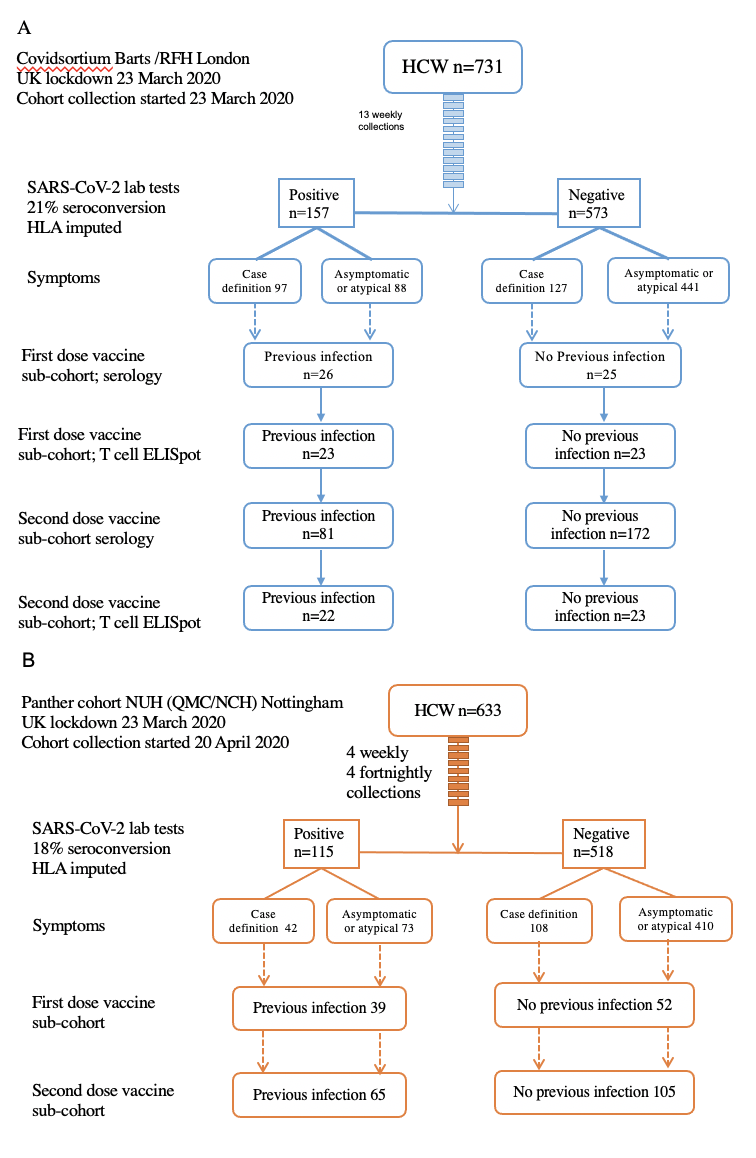


Supplementary Figure S2. DRB1 alleles and (A) seropositivity in HCW; (B) presence of case definition COVID-19 symptoms among seropositive healthcare workers. Association is defined as the correlation coefficient (beta) from logistic regression. For (**A**) IgG to N or spike seropositivity 1 or 0 being the outcome and carriage of DRB1 alleles the predictive variable. For (**B**) all individuals are seropositive and the outcome is presence of COVID-19 case definition symptoms. DRB1*13:02 is highlighted in blue.


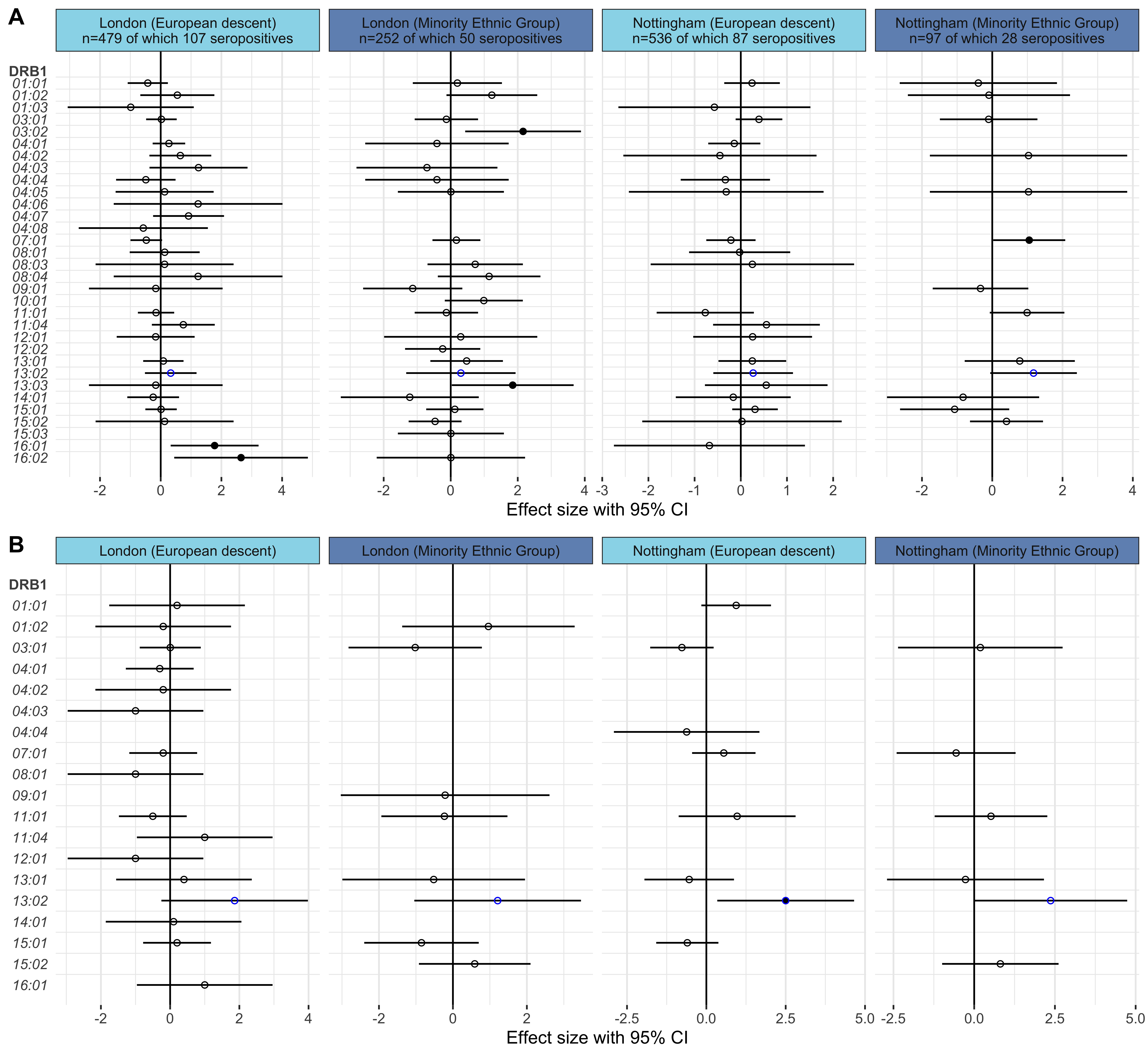


Supplementary Figure S3. Cumulative magnitude of the T cell response to spike (red) and N (blue) protein ordered by increasing magnitude of response in HCW with laboratory-confirmed SARS-CoV-2 infection (n=70). (A) HCW expressing one or two DRB1*15:02 (B) HCW expressing one or two DRB1*15:01.

Supplementary Figure S4. Association between DRB1 alleles and serological responses (IgG to spike S1) in COVIDsortium (London) and PANTHER (Nottingham) cohorts combined meta-analyses: (A) SARS-CoV-2 naïve HCW single dose vacinees (n=78); (B) HCW single dose vaccinees with prior SARS-CoV-2 infection (n=64); (A) SARS-CoV-2 naïve HCW single dose vacinees (n=78). DRB1*03:01 (red circle) associated with a fold increase in spike SI IgG responses and DRB1*04:04 and DRB1*07:01 (blue circle) associated with a fold decease in spike S1 IgG responses.

**A**

**B**
